# Constitutive and Plastic Gene Expression Variation Associated with Desiccation Resistance Differences in the *Drosophila americana* Species Group

**DOI:** 10.3390/genes11020146

**Published:** 2020-01-30

**Authors:** Jeremy S Davis, Leonie C Moyle

**Affiliations:** Department of Biology, Indiana University, Bloomington, IN 47405, USA; lmoyle@indiana.edu

**Keywords:** desiccation, RNA-seq, *Drosophila*

## Abstract

Stress response mechanisms are ubiquitous and important for adaptation to heterogenous environments and could be based on constitutive or plastic responses to environmental stressors. Here we quantify constitutive and plastic gene expression differences under ambient and desiccation stress treatments, in males and females of three species of *Drosophila* known to differ in desiccation resistance. *Drosophila novamexicana* survives desiccation trials significantly longer than the two subspecies of *Drosophila americana*, consistent with its natural species range in the desert southwest USA. We found that desiccation stress reduces global expression differences between species—likely because many general stress response mechanisms are shared among species—but that all species showed plastic expression changes at hundreds of loci during desiccation. Nonetheless, *D. novamexicana* had the fewest genes with significant plastic expression changes, despite having the highest desiccation resistance. Of the genes that were significantly differentially expressed between species—either within each treatment (>200 loci), constitutively regardless of treatment (36 loci), or with different species-specific plasticity (26 loci)—GO analysis did not find significant enrichment of any major gene pathways or broader functions associated with desiccation stress. Taken together, these data indicate that if gene expression changes contribute to differential desiccation resistance between species, these differences are likely shaped by a relatively small set of influential genes rather than broad genome-wide differentiation in stress response mechanisms. Finally, among the set of genes with the greatest between-species plasticity, we identified an interesting set of immune-response genes with consistent but opposing reaction norms between sexes, whose potential functional role in sex-specific mechanisms of desiccation resistance remains to be determined.

## 1. Introduction

Species employ many evolutionary strategies to survive pressure imposed by environmental stressors. Research on the resulting environmental adaptations often focuses on constitutive phenotypic differences between individuals exposed to different environments. However, it has long been recognized that adaptive traits can manifest as plastic phenotypes that vary dynamically within an individual, including over short periods of time [1]. Baldwin and later Simpson proposed that individuals with plastic phenotypes may be more likely to survive new environmental stressors compared to non-plastic individuals, and that this adaptive plasticity could be heritable [2,3]. Baldwin and others further described outcomes for the evolution of plasticity—including selection for increased adaptive plasticity in heterogenous environments, or a loss of plasticity due to either stability of a novel environment (genetic assimilation) or in order to prevent further phenotypic change (genetic compensation) [2,3,4,5,6]. Each of these scenarios is understood to be potentially important for adaptation to novel environments, but the mechanistic basis of trait plasticity and how it is shaped by natural selection across time, species, and environment remains an area of active and ongoing research. 

One contemporary approach to these questions involves examining gene expression responses to environmental manipulation. Changes in gene expression are a potentially efficient way for individuals to express short term plastic responses to acute environmental stress, as they can be both rapid and—when adaptive—tailored to meet the specific biological demands of environmental perturbations. Thermal tolerance has been a particular focus of many such studies, and these have uncovered evidence for adaptive plasticity between populations based on divergent natural thermal optima [7,8,9], experimental evolutionary change over several generations, and exposure to different developmental temperatures, in a wide variety of organisms [10,11]. In *Drosophila*, RNA-seq studies have assessed evidence for adaptive plasticity in heat stress tolerance [12,13,14,15], but also in other environmental stress responses including ethanol stress tolerance, and cold acclimation [16,17,18,19]. These studies have generated a wealth of information on genes and gene ontology (GO) groups that respond plastically to critical environmental stimuli. RNA-seq studies of this kind therefore provide a framework for addressing foundational questions about the nature of plastic gene expression responses to stress stimuli, including whether observed adaptive differences might be underpinned by these dynamic gene expression changes. 

Desiccation resistance is one such trait that is observed to differ between genotypes with different selective histories of exposure to xeric environments. Although an essential trait for preventing lethal water loss amongst all insects, variation among lineages in their degree of desiccation resistance also likely represents adaptive variation due to differential selection imposed by desiccating environments. Indeed, in *Drosophila* it has been shown that desiccation resistance can vary substantially among different populations and genotypes within species [20,21,22,23] as well as between species [24,25,26], and this variation is associated with differences in abiotic environmental conditions. Analyses aiming to identify genes and gene networks responsible for differences in desiccation resistance (reviewed [27,28]) have revealed this trait may be quite complex, with candidate genes associated with a wide array of functions ranging from stress-response and osmoregulation to insulin signaling and cuticular hydrocarbon (CHC) biosynthesis. Despite this interest in mechanisms of desiccation resistance, however, relatively few studies have looked at expression responses to direct desiccation stimuli among natural *Drosophila* populations. Matzkin and Markow [25] found that *Drosophila mojavensis* exposed to desiccation stress had large shifts in gene expression, and that many genes with the highest differential expression were related to metabolic regulation. Clemson et al. 2018 [29] used RNA-seq to examine genes responsible for varied desiccation survival amongst tropical and temperate Australian *D. melanogaster*, and found plastic gene expression that differed between populations at various timepoints of desiccation stress. Rajpurohit et al. 2013 [20] examined transcriptional differences among different populations of *D. mojavensis* from different geographical regions and host plants, and found that gene expression had population- and substrate-specific responses to desiccation stress at different time points. While each of these studies focused on intraspecific variation, closely-related species that differ in their desiccation resistance could similarly be used to evaluate the nature and importance of plastic gene expression responses to this important environmental stress. Contrasting gene expression in lineages with divergent natural histories and known differences in desiccation resistance allows an assessment of both their specific gene expression responses to this environmental stressor, and whether constitutive versus plastic responses might be more important in shaping this abiotic adaptation. 

Species in the *Drosophila americana* subgroup are one such group known to differ both in their abiotic habitats and in their physiological and phenotypic traits [26,30,31]. *Drosophila novamexicana* has a range limited to the arid southwest of the USA, while the two subspecies of *Drosophila americana* range a wide area east of the Rocky Mountains that includes a broad range of habitats that have greater volume and consistency of rainfall and moisture availability. Consistent with differences in historical natural selection imposed by habitat differences, desiccation resistance varies among these species, with *D. novamexicana* having a significantly higher desiccation survival time than both subspecies of *D. americana* [26]. Nonetheless, the mechanistic basis of desiccation resistance responses between species is not known, including the degree to which it reflects constitutive physiological variation versus a dynamic plastic response to ambient desiccating conditions, and whether gene expression variation might be responsible for plastic components of this variation. Here we quantify the nature of gene expression changes that are associated with exposure to acute desiccation stress, by examining whole-body RNA expression levels in males and females from one population of each species, at 0 and 3-hour time points of exposure to desiccation stress. Our goals were to (1) assess the level of baseline gene expression divergence between species; (2) assess the nature and pattern of gene expression responses to acute desiccation stress (their gene expression reaction norms); and (3) evaluate whether plasticity in gene expression between species reflects a pattern of genome-wide expression changes to desiccation stress or if responses are more focused in individual genes or pathways. 

## 2. Materials and Methods 

### 2.1. Experimental Lines and Desiccation Treatment

Fly lines used for each species were isofemale lines obtained from the National *Drosophila* Species Stock Center (NDSSC), now housed at Cornell (http://blogs.cornell.edu/drosophila/). The *Drosophila novamexicana* stock was originally collected in San Antonio, NM (stock number: 15010-1031.08), the *D. americana americana* stock is from Chadron, NE (15010-0951.06), and the *D. a. texana* stock is from Jamestown, SC (15010-1041.29). All fly stocks were reared on standard cornmeal media prepared by the Bloomington *Drosophila* Stock Center (BDSC) at Indiana University, and were kept at room temperature (~22 °C), prior to the experiment.

Acute desiccation resistance was previously assessed in nine different strains of *D. americana* group species (three strains per species) including the three focal strains examined in this study and found to be elevated in both males and females of *D. novamexicana* [26]. To do so, individual flies were placed in desiccating glass vials where they were observed until death (full details for this assay are available in Davis and Moyle [26], and see also below). Using the data from Davis and Moyle, we re-analyzed survival times under desiccation using ANOVA with species and sex as independent variables, in order to quantify differences in desiccation resistance between the three focal strains examined here. 

Male and female flies used during the experiment were virgins collected and isolated by sex within 24 h of eclosion using light CO_2_ anesthetization. Both sexes were then raised for 7 days on cornmeal media as above in order to reach maturity. On the 7th day, flies were exposed to one of two treatments: Desiccation treatment flies were aspirated to individual modified *Drosophila* culture vial which contained a layer of 20 g of Drierite, 10 g of desiccated cork, a piece of cheesecloth, and sealed by a layer of parafilm, while control flies were transferred to similar vials that lacked the cork and Drierite. The experimental set up was identical to the desiccation treatment used in Davis and Moyle in order to induce the same response as the observed phenotype [26]. After 3 h, individuals from both treatment groups were flash frozen using liquid nitrogen and transferred to −80 °C freezers to await RNA extraction. The three-hour desiccation stress treatment was chosen as an intermediate time-point that would to allow sufficient exposure to elicit a gene expression response to acute desiccation stress while avoiding gene expression changes associated with the onset of death. Two general reference points were used in this determination: prior studies of gene expression responses to desiccation in other *Drosophila* species, and prior data on responses to desiccation in our focal species. First, although studies in other species differ in desiccation methods and natural phenotypic variation in desiccation responses, exposure to a more mild desiccation stress has been shown to be sufficient to elicit gene-expression responses within 2.5 h in *Drosophila melanogaster* [32]. Second, in our previous analysis, the earliest observed mortality in individual desiccation trials began just prior to 6 h after initiating desiccation exposure (Davis and Moyle 2019). Together, these indicate that 3 h should be sufficient to capture gene expression responses to desiccation while avoiding gene expression of individuals close to death, under our experimental conditions.

### 2.2. RNA Pooling, Extraction, and Sequencing

We performed RNA extractions for 3 biological replicates of each sex, species, and treatment group for a total of 36 samples; to obtain sufficient RNA, each biological replicate contained a pool of 20 individually frozen flies. RNA extractions were performed using a hand homogenizer followed by RNeasy mini kit purification with DNase treatment (Qiagen, Hilden, Germany). RNA purity and concentration were checked by NanoDrop ND-1000 spetrophotometer (NanoDrop Technologies, Wilmington, DE, USA) and found to be equivalent across samples. RNA samples were then run on Illumina NextSeq with 150 bp paired-end reads. Each sample had between 8 and 15 million reads, except for a single biological replicate of male *D. americana* under control (ambient) exposure that contained fewer than 5 million reads—this sample was excluded from all additional analyses. Raw reads are available at NCBI Bioproject PRJNA602308. 

### 2.3. Read Mapping and Differential Expression Analysis

We followed much of the “new tuxedo” protocol from Pertea et al. 2016 [33] to map and assemble raw RNA-seq data into readcount data used for differential expression (DE) analysis. Because none of our target species have high-quality reference genomes, we mapped reads from each sample to the closest reference genome, *Drosophila virilis* (MRCA ~3 MYA [34]) (genome r1.7, from FlyBase: www.flybase.org), using HISAT2 [35]. We used an updated gene annotation for *D. virilis* from 2018 [36] to facilitate read mapping to as many known transcripts as possible. While mapping to an outgroup can reduce mapping success, particularly at highly divergent/rapidly evolving loci, using *D. virilis* for reference-based mapping for all of our species is preferable for at least two reasons: First its high-quality genome allows us to take advantage of annotation information when considering specific gene identification. Second, it avoids mapping bias among our samples because *D. virilis* is equidistantly related to each of our species. An ANOVA comparing the proportion of reads mapped in each sample (which varies between 74% and 88%) showed that mapping proportion does not differ between species (*F*(2) = 0.197; *p* = 0.822), or based on library size (*F*(2) = 0.042, *p* = 0.839), consistent with the expected lack of detection bias. Following read mapping, we used StringTie [37] to assemble and merge transcripts to create a uniform set of transcripts to compare abundance statistics across groups. Abundance was quantified as minimum mean counts per million (CPM). Note that, although transposable elements (TEs) have also been proposed as a potentially important mechanism of stress response [38], because the *D. virilis* genome currently lacks complete TE annotation information, we did not investigate TE expression differences between species or their influence on plastic responses to stress in this study. 

All subsequent analyses were performed on these read CPM data using R version 3.4.3. To determine which genes were differentially expressed (DE) within each of the control and desiccation treatment datasets, for each gene (transcript) we ran an ANOVA on CPM with sex, species, and the interaction of sex and species as independent variables. The interaction term identifies the genes whose sex-specific expression differs between species. For this analysis, genes were considered DE if *p* < 0.05 after Benjamini–Hochberg correction to control for multiple testing. Genes identified as DE from this analysis were passed to a Gene Ontology (GO) analysis (see below). Additionally, because this analysis indicated that there were fewer genes DE in the desiccation treatment relative to the control (see Results), we also evaluated whether gene expression variance among samples was higher in the desiccation treatment versus the control treatment. To do this, for each gene, we computed the log-transformed variance among samples within each of the control and desiccation treatments. We then performed a paired *t*-test to compare variances between the two treatments across all genes and evaluate which treatment had the larger variance between samples. 

Within each sex, for each species we defined a gene as plastic for desiccation stress if it had a greater than 2-fold difference in mean expression between the ambient and desiccation treatments, regardless of whether it was significantly DE between species or sexes within each treatment (above). This gave us a set of plastic genes for each species and sex that could be passed to a GO enrichment analysis (below). We also aimed to determine the genes that showed the largest differences in plastic response to desiccation stress between species; however, because individual samples are not paired between desiccation and control treatments, we must use average fold-changes for each species and sex, ruling out tests of variance such as ANOVA. Instead, we identified the transcripts with the largest variance in plastic fold-change expression between species, within each sex. For each of the top 20 genes that had the highest variance in plasticity between species within each sex, we identified their putative function either by FlyBase matches or BLAST (see below). 

### 2.4. Functional Identification and Enrichment 

For all functional annotations as well as GO enrichment analyses, we used FlyBase matches to *D. melanogaster* annotated orthologues, as *D. melanogaster* has more complete and specific functional annotations, including GO annotations, compared to *D. virilis* [18,39]. To identify the putative function of DE genes in our dataset, we identified functional classification using the GO enrichment tool on FlyMine with a Benjamini–Hochberg correction with False Discovery Rate (FDR) set at 0.05 (www.flymine.org, v.37). This GO enrichment analysis was performed separately for each set of genes identified as DE either between species or the sex by species interaction term from the ANOVA and repeated for both control and treatment datasets for a total of 4 separate analyses. We also performed this GO enrichment analysis separately for each set of plastic genes identified for each species and sex with greater than 2 fold-change between treatments (6 total analyses).

For functional annotation of the set of genes with the greatest variance in gene expression response between species, we used FlyBase’s functional summaries—which include UniProt and InterPro analyses—or where absent, summarized identified gene group and protein family relationships. Some genes annotated in Yang et al. (2018) [36] do not have matches to *D. melanogaster* FlyBase genes, so we aimed to search for genes with similar protein products to evaluate function. We input fasta sequences for the transcript to BLAST, UniProt, and InterPro databases to identify genes with protein products related to the predicted product of these genes. 

## 3. Results

### 3.1. Drosophila novamexicana Have Greater Survival under Acute Desiccation Stress 

**Figure 1:**
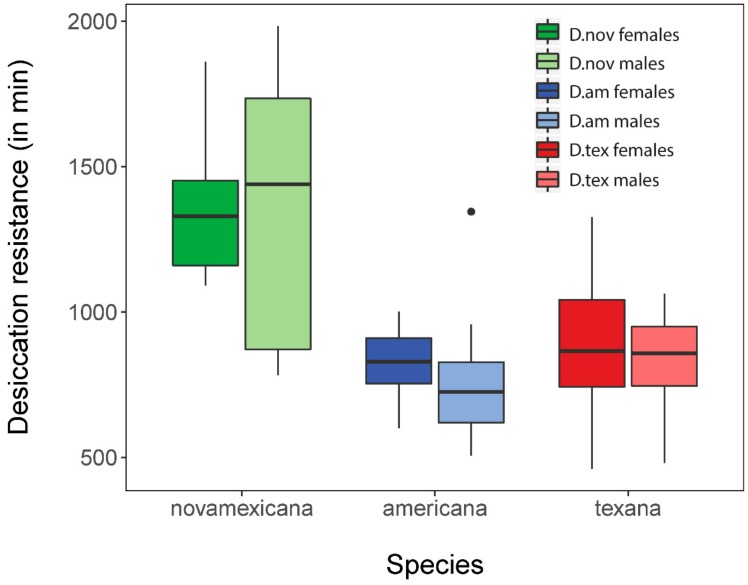
Variation in desiccation resistance between species and sexes, in minutes survived under acute desiccation (*n* = 5 for all identities) (means = black bars; quartiles = boxes). Original data is from Davis and Moyle 2019 [26]. ANOVA shows that species (F(2) *=* 10.16; *p* = 0.00027) but not sexes (F(1) *=* 0.66; *p* = 0.42) significantly differ in desiccation resistance.

Using data from Davis and Moyle [26], we performed an analysis of differences in desiccation resistance survival between the three populations used in this study. *D. novamexicana* males and females showed the highest survival, with average survival time of 1345.3 and 1367.7 min, respectively. *D. americana* subspecies did not survive as long, with male and female *D. a. americana* surviving for 820.6 and 767.2 min, respectively, and *D. a. texana* males and females living on average 827.4 and 883.6 min (Figure 1). ANOVA revealed that species significantly differed in survival time (F(2) = 10.16; *p* = 0.00027) but there was no difference between sexes (F(1) = 0.66; *p* = 0.42). This result agrees with our previous inferences and with expectations based on habitat differences between these species [26]. In particular, because *D. novamexicana* is found primarily in xeric (desert) regions of southwestern USA, elevated desiccation resistance in this species is consistent with adaptation to reduced moisture availability throughout its natural range.

### 3.2. Gene Expression Variation Is Greater under Ambient Than Desiccating Conditions

We obtained full-body RNA transcript data for three replicates of each sex, within each of three species, for both treatments (control and 3-hour desiccation), for 36 total samples. Each sample had between 8 and 15 million reads, except for a single biological replicate of control male *D. americana*; this sample was excluded from further analysis. After filtering out genes with low transcript abundance across all remaining samples, we retained 11,301 unique genes to evaluate differential expression (DE) in either control or desiccation treatments. 

To assess general patterns of gene expression variation, we first examined this dataset to determine the number of DE genes between species and sexes within each treatment. In the control treatment (no desiccation stress), after FDR correction 7558 (67% of our 11301 total transcripts) were DE between sexes, 284 (2%) DE between species, and 32 (<1%) had a significant interaction between species and sex, with 7659 unique DE genes between any category. In the desiccation treatment, there were fewer overall genes with DE between sexes (2483, 22%), species (58, <1%), or interactions between sex and species (31, <1%) for a total of 2514 unique DE genes (summarized in Table 1). 

The observation that fewer genes are DE under desiccation stress compared to under non-desiccating conditions could be explained by two alternative scenarios. First, acute desiccation stress might reduce the number of gene expression differences between groups if this treatment elicits genome-wide stress responses that are conserved across sexes and species, so that gene expression is more similar between these groups compared to under ambient conditions. Alternatively, the imposition of desiccation stress might stochastically elevate variation in gene expression among samples, and thereby reduce our power to detect species and sex differences specifically within this treatment. Under the latter scenario, we would expect variance in gene expression between samples for a given gene to be higher in the desiccation treatment compared to the control/ambient treatment. A paired t-test of log-transformed variance among samples across all gene revealed that the control samples actually had higher variance than samples from the desiccation treatment (*t*(11300) = 13.5, *p* < 0.001). This observation suggests that desiccation stress elicits a common set of gene expression responses across both sexes and species, resulting in fewer expression differences between groups specifically during this stress treatment. This is exemplified by the substantial number of genes (252) that were DE between species under the control (ambient) treatment, but that did not show species-specific differences during exposure to desiccation stress. These observations might indicate that desiccation resistance variation between species is not due to differences in broad genome-wide expression responses to stress, but rather is underpinned by allelic variation and/or plasticity within a more narrow set of genes. Interestingly, when we examined the genes that showed significant DE between species within each treatment (control and desiccation) we also found no evidence for enrichment of any specific Gene Ontology (GO) categories, either in genes DE among species or the interaction between species and sex. These results similarly indicate that the set of genes that differ between species within either treatment do not reflect generalized changes in broad physiological GO groups.

### 3.3. Many Differentially Expressed Genes Show Constitutive Differences between Sexes and Species, Regardless of Treatment 

To identify DE genes that differ in constitutive expression regardless of treatment effects, we examined the set of genes that were found to be DE for sex, species, or their interaction, in both control and desiccation treatments. This set of genes represents those that have constitutive differences between sexes or species regardless of the presence/absence of desiccation stress, and therefore the set of genes with non-plastic expression differences that could contribute to phenotypic differences between groups. We found that 2479 of the 2483 genes (99.8%) DE between sexes during desiccation stress were also DE in the control (ambient) treatment. The observation of many differences in whole body gene expression between sexes regardless of stress condition is consistent with previous work in other *Drosophila* species [25], and is likely due to general sex-specific differences in metabolism, reproductive biology, or other processes that have little to do with stress response. Note that this inference, combined with our general finding that there are many more gene expression differences between sexes than between species, lead us to perform all subsequent analyses on separate male and female datasets. 

Similar to sex differences, we found that 32 of the 58 genes that were DE between species during the desiccation treatment were also among the 284 that differed between species in the control group, and that nine genes with a significant interaction between sex and species were in common between the 31 and 32 genes DE in the desiccation and control treatment groups, respectively. In general, these data indicate that a substantial proportion of genes that are differentially expressed between species and between sexes under desiccation stress are constitutive differences also observed in the absence of desiccation stress. While these global patterns suggest a potentially important role for constitutive gene expression variation in species differences in desiccation resistance, they also support a potential role for desiccation-specific gene expression responses. For example, of the 54 genes DE between species during desiccation stress, the remaining 26 genes had species-specific responses that were only observed under acute desiccation stress. These loci indicate there is variation among species specifically in their plastic gene expression responses (their expression ‘norms of reaction’) to desiccation stress, an observation that we aimed to address more directly with analyses of gene expression plasticity below.

### 3.4. Hundreds of Genes Show Plasticity in Response to Desiccation Stress within Each Species

**Figure 2:**
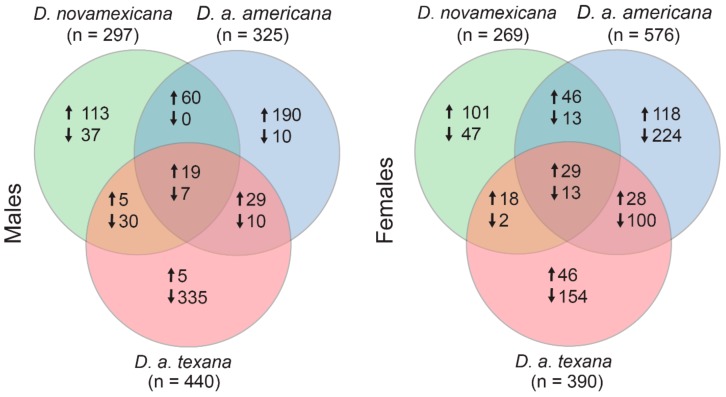
Numbers of genes that are shared versus species-specific in their plastic gene expression responses to desiccation stress (log_2_ fold change or greater), within males (top) and females (bottom). Total number of plastic genes for each species indicated in parentheses. Arrows indicate whether gene expression is elevated (up) or reduced (down) in the desiccation treatment, relative to the control/ambient treatment.

Using the criterion of a log_2_ fold-change difference in expression between desiccation and control treatments, we identified the set of genes that showed a plastic response to desiccation stress within each species for each sex. Similar to previous intraspecific studies of gene expression responses to desiccation [20,25,29], we found that a large number of genes differed in expression between ambient and desiccating conditions, and numerous of these changes were lineage specific. Figure 2 shows the numbers of genes that are shared versus species-specific in their plastic gene expression responses to desiccation stress, within each sex. For males, *D. novamexicana* showed plastic expression in 271 genes (197 up, 74 down), *D. a. americana* had 325 plastic genes (298 up, 27 down), and *D. a. texana* had 440 (58 up, 372 down) for a total of 826 unique genes with plastic expression in at least one species. Females had similar number of overall plastic gene expression changes—with 269 (194 up, 75 down) genes in *D. novamexicana*, 576 (226 up, 350 down) in *D. a. americana*, and 390 (118 up, 272 down) in *D. a. texana*, for a total of 938 unique plastic genes. 

**Figure 3:**
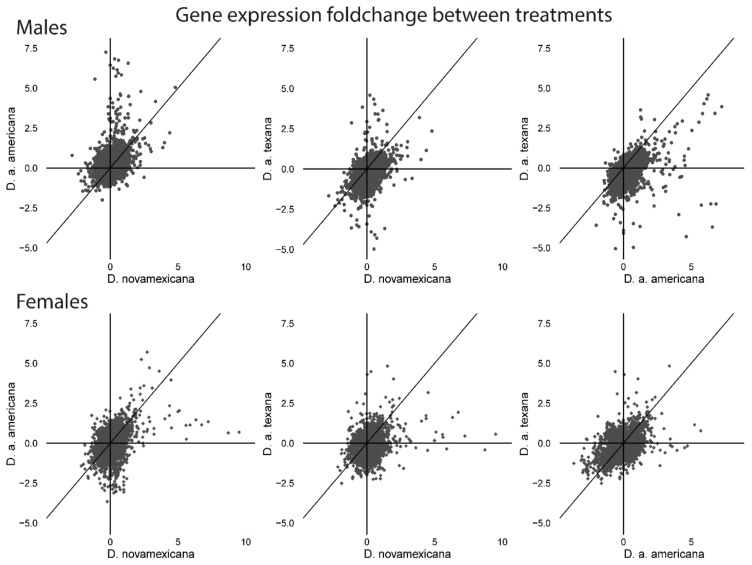
Pairwise species comparisons of (log_2_ fold) gene expression differences between ambient (non-desiccating) and desiccation conditions, for males (top) and females (bottom). Each point shows the expression change for one gene for each pair of species; diagonal lines are y=x and represent the expectation if species share an identical plastic response to desiccation stress. Points that lie far from this line indicate genes that have greater differences in plastic expression patterns between species.

**Figure 4:**
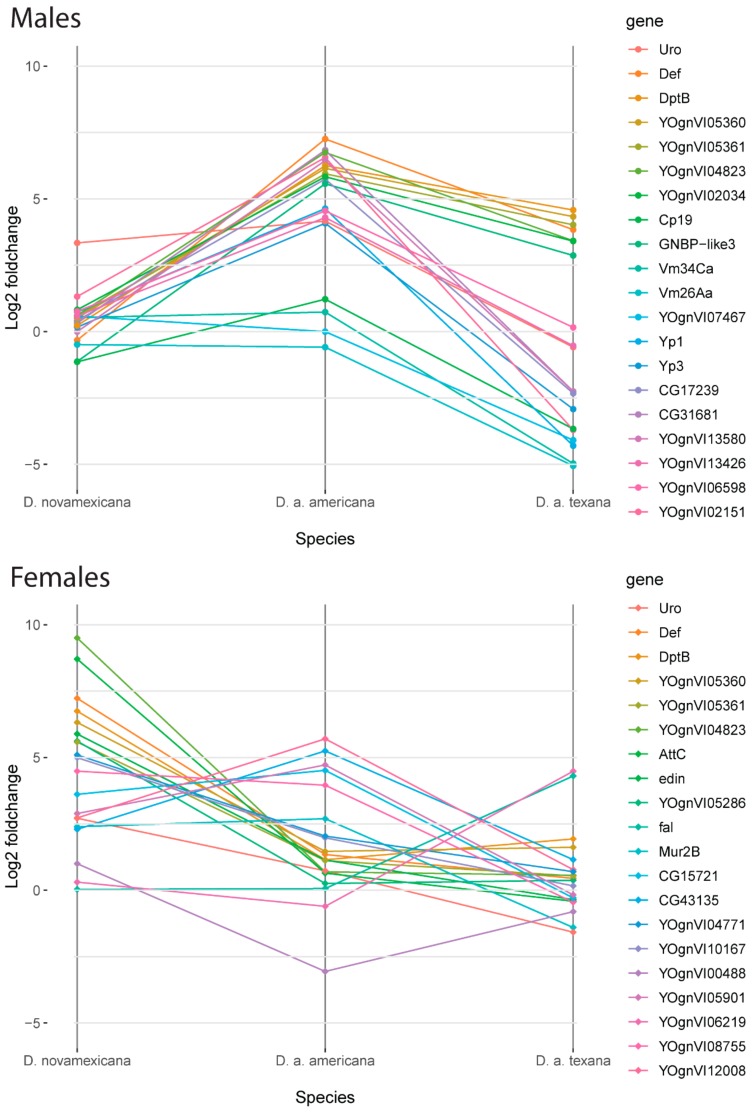
Top 20 genes by greatest between-species variance in fold-change for males (top) and females (bottom). Six genes: *Uro, Def, DptB, YOgnVI05360, YOgnVI05361*, and *YOgnVI04823* are shared between males and females, and all but *Uro* show opposing patterns between the sexes. Functional information about these genes is given in Table 2 and Table 3.

A number of interesting observations emerge from these data. First, a GO analysis to assess if genes that were plastic (>log_2_ fold-change) between treatments were enriched for broader functional categories within each sex and species, found that no group had significant enrichment for any GO categories after Benjamini–Hochberg FDR correction. This differs from Matzkin and Markow’s [25] analysis within *D. mojavensis* where they found genes with expression shifts under desiccation were frequently related to metabolic regulation. Instead, our result suggests that if gene expression changes are responsible for phenotypic desiccation resistance differences between species, these expression responses are not concentrated within specific functional classes of genes. Second, for both males and females, the species with the highest desiccation resistance—*D. novamexicana*—actually has the fewest number of genes with plastic (log_2_ fold-change) responses to desiccation stress (Figure 2). Similar to our inference from the within treatment analyses (above), this again suggests that—if gene expression plasticity underlies increased desiccation resistance—it is not conferred by numerous, genome-wide expression changes, but instead involves a more targeted or specialized response to desiccation stress at fewer genes. Finally, some patterns of expression plasticity appear to differ between species depending upon sex. Figure 3 shows the pairwise relationships between plastic expression changes in each gene, separately for each sex. For males, the magnitude of expression plasticity is most modest in D. novamexicana males, where the total fold-change range of plastic responses varies between ~2.81 and 4.80, compared to D. a. americana (range: ~2 to 7.25), and D. a. texana (range: ~5.06 to 4.58). Additionally, the most plastic genes in D. a. texana males also tend to be the most plastic in D. a. americana (Figure 3 top right), although these expression changes are not always in the same direction, as some of the most up-regulated plastic genes in D. a. americana are substantially down-regulated in D. a. texana. (Both of these patterns can also be seen in the top 20 most plastic genes among males; see further below, and Figure 4). Interestingly, this pattern is different in females, where D. novamexicana females have the broadest range of plastic expression (~2.13 to 9.50 fold-change) compared to D. a. americana (~3.66 to 5.70) and D. a. texana (~2.52 to 4.8). Females also do not show the same dichotomy of opposing plastic expression patterns in some genes that is between the two americana subspecies males. 

### 3.5. Genes with the Largest Differences in Plasticity between Species Reveal a Sex-Specific Pattern of Gene Expression Response to Stress

To further explore the behavior and identity of the genes with the largest plastic responses, we identified the top 20 genes that had the greatest between-species variance in plastic response to desiccation stress (measured as fold-change between treatments) within each sex (Figure 4; Table 2 and Table 3). In both males and females, we found that the majority of these genes have previously described functions associated with immune-responses—particularly bacterial defenses—and with reproductive processes such as egg and sperm production, based on functional annotations from *D. melanogaster* (see Methods). Our observation that several vitelline membrane and egg yolk genes are among those that have the highest variance in among-species plasticity specifically in males is particularly interesting, as these genes are primarily thought to be important for female fertility. Nonetheless, while earlier studies indicated that some of these genes were female-specific (e.g., Vm26Aa in *D. melanogaster* [40]), more recent analyses also indicate detectable expression in males. For example, the modENCODE expression analysis indicates that Vm26Aa and Vm34Ca are both expressed in male flies at low but detectable levels, and that Yp1 and Yp3 yolk protein genes have relatively broad expression in males [41]. Moreover, all four of these genes have been shown to have elevated expression under cold- heat- and ethanol stress [42]—suggesting that their functions extend beyond female reproductive functions, and are associated broadly with stress response. Intriguingly, within our dataset these plastic gene responses appear to be sex-specific. Although all four genes have large differences in plasticity among males of different species, female gene expression remained both relatively non-plastic (average change in expression for all species/genes: ~0.149) and had low variance between species (average variance: 0.150). Prior analyses indicate that, for example, the yolk proteins Yp1 and Yp2 are downstream of sex determination pathways that alter expression patterns, and are associated with sex-specific effects on longevity [43]. Together with these previous observations, our data also indicate that these genes have broader functions than just reproductive success, and that these functions might differ between sexes.

In addition, a consistent pattern emerged across several genes with a shared sex-specific pattern of expression. For males, 6 of the top 30 highest variance genes (Def, DptB, YOgnVI02304, YOgnVI05360, YOgnVI05361, YOgnVI04823, and YOgnVI04771) all show relatively little plastic expression within *D. novamexicana*, but substantial upregulation in both *D. a. americana* and *D. a. texana* during desiccation stress. In females, six of the same genes (Def, DptB, YOgnVI05360, YOgnVI05361, YOgnVI04823, and YOgnVI04771) are also within the top 30 highest variance genes except the expression patterns for all six of these, in addition to four others (AttC, edin, YOgnVI05286, and YOgnVI04823) are entirely unlike those observed in males: *D. novamexicana* females have plastic upregulation of these genes, while *D. a. americana* and *D. a. texana* show either no plastic expression or modest upregulation. Note that another ‘top 30’ gene for both males and females—Uro—also shows this behavior in females but the gene expression pattern is less consistent in males (Table 2). The observation of different plastic responses in each sex, depending on species, also matches that seen among general patterns in Figure 3. 

The reason why reaction norms of the most variable plastic genes behave differently in males vs. females, and why many of these genes are associated with immune-response functions, remains to be determined. Genes associated with immune response could be associated with abiotic stress responses if they are elicited as part of a broader reaction to stress stimuli. However, the very different reaction norms observed for the same genes in conspecific males and females—especially in *D. novamexicana*, where both sexes show equally elevated desiccation resistance (Davis and Moyle [26], and above)—make simple inferences about the adaptive function of plastic responses in these loci more challenging. One potential explanation for this discrepancy is that these genes have direct or indirect functional differences between the sexes that confer different but equivalently adaptive outcomes for males and females in response to desiccation stress. Alternatively, it might be that these genes—although strongly responsive to desiccation stress—are not functionally associated with adaptive responses to this stress. Stress responses are often associated with gene expression changes that have no effect or can sometimes be deleterious [44,45] These alternatives and implications of these expression changes can be explored in the future with more detailed analyses of these candidate loci. 

## 4. Conclusions

Here we aimed to characterize gene expression responses to desiccation stress among species that show adaptive differences in acute desiccation resistance. We found that desiccation stress generally reduced variance in gene expression between samples and reduced the overall number of differentially expressed genes between species, likely because a substantial fraction of gene expression under desiccation is due to generalized stress responses that are shared across all three lineages. Nonetheless, we also identified a smaller but significant set of genes with constitutive expression differences between species regardless of desiccation, and with unique species-specific responses to desiccation stress. This relatively modest number of genes with species-specific expression differences indicates that—if gene expression variation is responsible for differential survival under desiccation stress—this is likely based on a more limited set of loci with either constitutive or plastic gene expression responses, rather than broad genome-wide differences in gene expression among species. This inference is further supported by the observation that the most desiccation resistant species—*D. novamexicana*—was also the species with the fewest plastic expression responses to desiccation. While GO analyses identified no enrichment of functional groupings among either constitutive or plastic genes, both groups of loci are interesting candidates for further work on (non-sex-specific) species differences in desiccation resistance. Finally, among the genes with the largest between-species variance in expression responses, we identified a set of genes that appear to have consistent and opposing norms of reaction between sexes. The possible role of these loci in sex-specific adaptive responses to desiccation remains to be examined in the future.

## Figures and Tables

**Table 1:** Number of genes found to be significantly DE for each term of the ANOVA, analyzed separately for each treatment. ‘Overlap’ indicates number of genes that were found to be significantly DE in both treatments.

	Treatment		
	Control (0 h)	Desiccation (3 h)	Overlap
**Species**	7558	2483	2479
**Sex**	284	58	32
Species* sex	32	31	9

**Table 2:** Top 20 genes with highest between-species variance in plastic responses to desiccation stress in males, as measured by fold-change in expression between control and desiccation treatments. * Loci also found in top 20 genes for females (Table 3). The next 10 most plastic loci are listed in Supplement Table S1.

Gene	*D. novamexicana* Fold-Change	*D. a. americana* Fold-Change	*D. a. texana* Fold-Change	Flybase/BLAST Functional Inferences
Uro *	3.342	4.164	−0.584	Factor independent urate hydroxylase
Def *	−0.321	7.257	3.841	Activity against gram+ bacteria
DtB *	0.228	6.253	4.580	Activity against gram- bacteria
YOgnVI05360 *	0.510	6.149	4.333	DptA-like, activity against gram- bacteria
YOgnVI05361 *	0.584	5.946	4.023	DptA-like, activity against gram- bacteria
YOgnVI04823 *	0.610	6.749	3.421	Uncharacterized.
**YOgnVI02034**	0.823	5.835	3.409	Cec2A-like lytic activity against gram- bacteria.
**Cp19**	−1.137	1.222	−3.662	Chorion-associated protein
**GNBP-like3**	−1.125	5.571	2.867	Carbohydrate binding protein
**Vm26Aa**	−0.485	−0.584	−5.055	Vitelline membrane protein family
**Vm34Ca**	0.530	0.736	−4.969	Vitelline membrane protein family
**YOgnVI07467**	0.584	0	−4.087	Vm26Aa-like, vitteline membrane family
**Yp1**	0.736	4.632	−4.297	Yolk protein. Carboxyl-esterase/lipase
**Yp3**	0.169	4.082	−2.918	Yolk protein. Carboxyl-esterase/lipase
**CG17239**	0.415	5.745	−2.321	Serine protease, trypsin family
**CG31681**	0.321	6.835	−2.247	Serine protease, trypsin family
**YOgnVI13580**	0	6.442	−2.247	Chymotrypsin, serine protease
**YOgnVI13426**	0.584	4.297	−0.530	Seminase
**YOgnVI06598**	0.761	4.549	0.159	Collectin-11 like, microbial binding
**YOgnVI02151**	1.321	6.558	−3.700	No match

**Table 3:** Top 20 genes with highest between-species variance in plastic responses to desiccation stress in females, as measured by fold-change in expression between control and desiccation treatments. *Loci also found in top 20 genes for males (Table 2). The next 10 most plastic loci are listed in Supplement Table S2.

Gene	*D. novamexicana* Fold-Change	*D. a. americana* Fold-Change	*D. a. texana* Fold-Change	Flybase/BLAST Functional Inferences
Uro *	2.711655	0.731511	−1.58085	Factor independent urate hydroxylase
Def *	7.230337	1.343954	0.447459	Activity against gram+ bacteria
DptB *	6.746452	1.148863	1.932886	Activity against gram- bacteria
YOgnVI05360 *	6.322839	1.463947	1.616671	DptA-like, activity against gram- bacteria
YOgnVI05361 *	5.583351	1.115477	0.540568	DptA-like, activity against gram- bacteria
YOgnVI04823 *	9.503163	0.691878	0.561879	Uncharacterized
**AttC**	8.709945	0.643856	−0.41504	Activity against gram- bacteria
**edin**	5.885696	1.137504	−0.35614	Elevated during infection, humoral immune response
**YOgnVI05286**	5.616511	0.253119	0.373458	Immune-induced peptide
**fal**	0.030096	0.06286	4.303781	GTPase activity
**Mur2B**	2.393914	2.694849	−1.40439	Mutin, chitin-binding domain
**CG15721**	3.613117	4.515939	−0.28951	Uncharacterized
**CG43135**	2.294183	5.247928	1.152003	Uncharacterized
**YOgnVI04771**	5.095727	2.041027	0.698696	AttB-like, Activity against gram- bacteria
**YOgnVI10167**	4.993545	1.975044	0.164844	O-acyltransferase-like protein
**YOgnVI00488**	1	−3.05889	−0.80735	No match
**YOgnVI05901**	2.888313	4.719563	−0.17454	No match
**YOgnVI06219**	0.305847	-0.60638	4.483083	No match
**YOgnVI08755**	4.485427	3.955985	−0.4498	No match
**YOgnVI12008**	2.723741	5.702942	0.775039	No match

## Supplementary Materials

The following are available online at https://www.mdpi.com/2073-4425/11/2/146/s1, Table S1: Additional loci with high between-species variance in plasticity in males, Table S2: Additional loci with high between-species variance in plasticity in females.
