# Supplementary material for: Constitutive and Plastic Gene Expression Variation Associated with Desiccation Resistance Differences in the Drosophila americana Species Group
Source: Genes (Basel). 2020 Jan 30;11(2):146. doi: 10.3390/genes11020146 (PMC7073762; doi:10.3390/genes11020146)
Supplement: Supplementary file 1 [file genes-11-00146-s001.pdf]

Table S1. Additional loci with high between-species variance in plastic responses to desiccation stress in males, as measured by fold-change in expression between control and desiccation treatments. Extension of Table 2. Asterisk denotes also found in female top 20 list (Table 3).

Gene	*D. novamexicana* foldchange	*D. a. americana* foldchange	*D. a. texana* foldchange	Flybase/BLAST functional inferences
**Cp36**	0.321928	0	-3.90689	Chorion-associated protein
**YOgnVI09371**	0	3.115477	-1.45943	Uncharacterized
**CG43134**	-0.43296	2.260528	-2.37851	Uncharacterized
**YOgnVI04771***	0	4.353448	2.943711	AttB-like, Activity against gram- bacteria
**YOgnVI06452**	0.485427	3.115477	-1.1375	Uncharacterized
**Vm26Ab**	-0.73697	0.736966	-3.48113	Vitelline membrane protein family
**YOgnVI11671**	0	2.938599	-1.22239	Uncharacterized
**YOgnVI04392**	1.355134	4.482128	0.487121	No match
**YOgnVI13095**	0.558442	3.683526	-0.08746	No match
**YOgnVI07992**	0.415037	3.415037	-0.41504	Peptidase S1 domain protein

Table S2. Additional loci with high between-species variance in plastic responses to desiccation stress in males, as measured by fold-change in expression between control and desiccation treatments. Extension of Table 3. Asterisk denotes also found in male top 20 list (Table 2).

Gene	*D. novamexicana* foldchange	*D. a. americana* foldchange	*D. a. texana* foldchange	Flybase/BLAST functional inferences
**YOgnVI02157**	0.998	-2.906890596	-0.321928095	Uncharacterized
**CG3213**	0.978	-2.624490865	-2.222392421	Outer dense fibre protein 2-related
**YOgnVI03138**	2.484033592	3.598259323	-0.211054877	Glyco-protein domain
**YOgnVI14951**	-0.118877396	-0.456003549	3.092169844	Ras-GEF domain-containing protein
**YOgnVI06315**	2.209453366	3.071462363	-0.598186196	Alkaline-phosphatase domain
**YOgnVI02327**	-1.364996817	1.73940877	1.983511877	Heat-shock protein
**CG13083**	2.211334015	3.465058103	-0.138265238	Chorion-associated protein
**YOgnVI06033**	0.736965594	-2.906890596	-1.222392421	Uncharacterized
**YOgnVI02034***	4.523561956	0.959358016	3.169925001	Cec2A-like lytic activity against gram- bacteria.
**YOgnVI05608**	0.263034406	-1.874469118	1.68589141	Uncharacterized
